# An IBCLC in the Maternity Ward of a Mother and Child Hospital: A Pre- and Post-Intervention Study

**DOI:** 10.3390/ijerph120809938

**Published:** 2015-08-20

**Authors:** Antonella Chiurco, Marcella Montico, Pierpaolo Brovedani, Lorenzo Monasta, Riccardo Davanzo

**Affiliations:** 1 Division of Neonatology and NICU, Institute for Maternal and Child Health—IRCCS “Burlo Garofolo”, Trieste, TS-34137, Italy; E-Mails: ant.chiurco@gmail.com (A.C.); pierpaolo.brovedani@burlo.trieste.it (P.B.); riccardo.davanzo@burlo.trieste.it (R.D.); 2 Clinical Epidemiology and Public Health Research Unit, Institute for Maternal and Child Health—IRCCS “Burlo Garofolo”, Trieste, TS-34137, Italy; E-Mail: marcella.montico@burlo.trieste.it

**Keywords:** International Board Certified Lactation Consultant, breastfeeding promotion, mother and child hospital

## Abstract

Published evidence on the impact of the integration of International Board Certified Lactation Consultants (IBCLCs) for breastfeeding promotion is growing, but still relatively limited. Our study aims at evaluating the effects of adding an IBCLC for breastfeeding support in a mother and child hospital environment. We conducted a prospective study in the maternity ward of our maternal and child health Institute, recruiting 402 mothers of healthy term newborns soon after birth. The 18-month intervention of the IBCLC (Phase II) was preceded (Phase I) by data collection on breastfeeding rates and factors related to breastfeeding, both at hospital discharge and two weeks later. Data collection was replicated just before the end of the intervention (Phase III). In Phase III, a significantly higher percentage of mothers: (a) received help to breastfeed, and also received correct information on breastfeeding and community support, (b) started breastfeeding within two hours from delivery, (c) reported a good experience with the hospital staff. Moreover, the frequency of sore and/or cracked nipples was significantly lower in Phase III. However, no difference was found in exclusive breastfeeding rates at hospital discharge or at two weeks after birth.

## 1. Introduction

International health agencies and pediatric societies recommend exclusive breastfeeding (BF) for six months and continuation of partial BF for more than 12 or 24 months [1,2]. In fact, overwhelming epidemiological evidence exists that breastfeeding has many health benefits for both the mother and the child [3,4]. Successful breastfeeding is related to multiple influencing factors which are personal, cultural, socio-demographical, and economical in nature [5]. Strategies and interventions to foster mothers to start and maintain breastfeeding consequently need to take these factors into account [6]. In addition, successful breastfeeding is significantly influenced by a good start at birth, which also depends on hospital organization, quality of care for newborn infants, and support received by new mothers [7].

Despite the evidence, international data on breastfeeding rates at birth are not yet satisfactory for many industrialized countries, such as Italy [8], France [9], and Ireland [10], calling for a series of multiple and integrated interventions to promote breastfeeding [11], among which we should mention those provided by the lactation consultant [12]. International Board Certified Lactation Consultants (IBCLCs) have heterogeneous backgrounds, and operate in different kinds of health facilities (hospitals, clinics) and private homes where support and management of breastfeeding are requested. There are currently over 27,450 IBCLCs worldwide, operating in 101 countries (http://www.iblce.org; accessed on 27 July 2015). In the course of their work, IBCLCs may help women with breastfeeding as part of other health care duties, or they may just assist women with breastfeeding. The IBCLC is a professional with great knowledge and skill regarding breastfeeding who can spend additional time with parents who are in need [13]. In the context of IBCLCs’ work, ethics are particularly important as the work implies intimate contact with women at a vulnerable time in their lives, access to private information, and having to deal with medical problems and dilemmas [14]. Evidence in the literature on the impact of IBCLC intervention in promoting breastfeeding is still relatively limited due to the new role and brief history of IBCLCs, different definitions of lactation consultant (LC) in available studies (IBCLC *vs.* differently qualified LC), and different settings of intervention such as Neonatal Intensive Care Units (NICUs) [15,16], military facilities [17], community health centers [18,19], and hospitals [20]. The process of integrating an IBCLC with the health staff has been described in an outpatient setting [21], and not yet in an in-patient service. We therefore conducted a study to evaluate the effects of the integration of an IBCLC for the support of breastfeeding in a maternity hospital environment.

## 2. Materials and Methods

The present study was conducted in the maternity ward of a mother and child research hospital (Institute for Maternal and Child Health, IRCCS “Burlo Garofolo”) in Trieste (Friuli Venezia Giulia region, Northeastern Italy), delivering approximately 1800 infants per year. In our maternity hospital, the 10 Steps of the Baby-Friendly Hospital Initiative (BFHI) [22,23] have not been fully implemented yet, even though skin-to-skin contact and first feed in the delivery room, on demand and unlimited rooming-in, no routine bottle feeds, and no pacifiers are recommended, although unwritten, hospital policies. Mothers are provided with breastfeeding support by regular nurses who are specifically trained with the 18- or 20-h United Nations Children’s Fund (UNICEF) and World Health Organization (WHO) course on breastfeeding in the first 12 months of their service with the rooming-in staff [24]. Babies of mothers who underwent cesarean section (CS) are sometimes attended by nurses in the first six to 12 h after childbirth, depending on the pattern of respiratory and cardiovascular adaptation to extrauterine life and maternal general conditions. More than 90% of mothers experience extensive rooming-in. However, mothers are free to ask for their baby to be cared for in the nursery for some time at night and during obstetrical visits and when using the toilet. As a consequence, around 61% of mothers practice continuous rooming-in from delivery to hospital discharge (unpublished data from a 2004 hospital-based survey).

The decision to administer nutritive liquids other than breast milk to newborn infants is made by doctors and nurses, referring to a protocol for the management of excessive neonatal weight loss, which considers the administration of formula for the prevention of hypernatremia [25,26,27]. If formula is prescribed, a bottle is always used. Pacifiers are sometimes used according to the decision of the mother and the special needs of the newborns (*i.e.*, twins, pain treatment during blood sampling in the absence of other analgesic approaches such as sucking at the breast, fussiness during phototherapy, not responding to frequent breastfeeding). In our maternity hospital, visual messages promoting formula feeding as well as prescribing formula to mothers exclusively breastfeeding at hospital discharge are prohibited. Giving milk formula samples to mothers is prohibited by a national law [28].

After three decades of slow and continuous progress in breastfeeding promotion inside the hospital, the monitoring of breastfeeding rates at discharge using WHO definitions (exclusive breastfeeding: no other food or drink, not even water, except breast milk (including milk expressed or from a wet nurse) for six months of life, but allowing the infant to receive oral rehydration salts (ORS), vitamins, minerals, and medicines; predominant: infant's predominant source of nourishment has been breast milk (including milk expressed or from a wet nurse as the predominant source of nourishment), but the infant may also have received liquids (water and water-based drinks, fruit juice), ritual fluids and ORS, drops, or syrups (vitamins, minerals, and medicines); full breastfeeding: exclusive breastfeeding plus predominant breastfeeding; complementary feeding: other foods and liquids (including formula) are introduced along with breast milk [27,28]) demonstrated a situation of stalemate in the years previous to the study (exclusive BF at 67%, 65%, and 66%, respectively, in 2003, 2004, and 2005), witnessing the need for a change in order to increase exclusive breastfeeding rates. The recall periods for the information on feeding status were: (a) from delivery to hospital discharge for data collected at childbirth, (b) previous 24 h for data collected after hospital discharge.

In February 2006, an IBCLC (ACh) was contracted for two years to support breastfeeding as an additional human resource to rooming-in nurse staff. The prospective study was divided into three phases. The 18-month intervention of the IBCLC (Phase II) was anticipated (Phase I) and followed (Phase III) by a survey collecting data on exclusive, predominant, and full breastfeeding rates (according to WHO definitions) and on factors influencing breastfeeding.

The research protocol (RC 14/06) was approved by the Review Board and by the Independent Bioethics Committee of the Institute for Maternal and Child Health, IRCSS “Burlo Garofolo”, Trieste, Italy.

### 2.1. Study Phases

#### 2.1.1. Phase I: Baseline

*Interview to mothers*. Mothers capable of understanding and speaking Italian were consecutively recruited in the hospital soon after delivery in the period 15 February–15 May 2006. All women were asked to sign a written informed consent form of adherence to the study. Inclusion criteria were: mothers of healthy term newborns, appropriate for gestational age, discharged from hospital from Monday to Friday. This choice allowed mothers to be recruited into the study by the IBCLC, who was also the main researcher (ACh), and whose service was six hours per day, Monday to Friday. At discharge, all recruited mothers were given an appointment for a telephone interview to be conducted two weeks later. A trained interviewer called the mother at the scheduled date. In case of no response, the mother was called during the following week until an answer was obtained. Each telephone interview included data on: (1) feeding in the previous 24 h, (2) previous BF experience, (3) attendance to antenatal BF courses, (4) skin-to-skin contact in the delivery room, (5) information received by mothers on the nutritional needs of their newborns in the first days of live, (6) breastfeeding during hospital stay and at discharge, (7) support to breastfeeding provided by hospital staff after childbirth, and, finally, (8) problems with breastfeeding encountered in the first two weeks after delivery, including sore and/or cracked nipples. Data provided by mothers were integrated with data obtained from clinical records, including type of delivery and feeding status at discharge from hospital, according to WHO definitions [30]. Information on feeding status was collected by the neonatologist and/or the nurse at the discharge visit from the feeding sheet of the clinical records, and mothers were asked to confirm.

#### 2.1.2. Phase II: Intervention

The IBCLC was appointed by the Hospital Director and her service initiated in the rooming-in ward in May 2006. Her role was to provide advice and practical support to mothers during their hospitalization and prepare them to continue breastfeeding following discharge. The service was provided five days a week, from 7 a.m. to 1 p.m. or from 12 a.m. to 6 p.m. The IBCLC provided individualized counseling, particularly to mothers after cesarean section (CS), prolonged and difficult labor, preterm delivery, and delivery of low-birth-weight infants (LBWI: <2500 g). At the beginning of each shift, the IBCLC was informed by the staff nurses regarding the mothers that were more in need, selected on the basis of breastfeeding problem acuity. On many occasions, however, the intervention of the IBCLC was directly requested by the mother. In principle, the IBCLC intervention was mostly second level, even if she could also take over some of the regular nurses’ responsibilities. The IBCLC also worked as liaison between the hospital and community health services. During the initial contact, which typically lasted 30 to 60 min, the IBCLC included an examination of the breast and of the appropriateness of the latching onto the breast. She also provided information on different topics such as frequent feeds, sore and/or cracked nipples, baby crying, and first mother-infant relationship, breast milk manual expression, or/and a pumping regimen, and she promoted the involvement of other family members. Mothers were encouraged to further contact the IBCLC by telephone if they had any questions or experienced any difficulty. A form was specifically prepared by the IBCLC to record her professional activities.

The IBCLC was directly supervised by the physician in charge of the research study (RD). During a preliminary meeting, the IBCLC was introduced to the nursing staff and her skills and tasks were illustrated. During the IBCLC intervention, periodic meetings were held with the participation of the IBCLC, the head nurse, the physician in charge of the study, and the Hospital Director.

#### 2.1.3. Phase III: Evaluation toward the End of the Intervention

On November 2007, 18 months after the beginning of the IBCLC intervention and while this intervention was still provided to mothers, a second series of telephone interviews was conducted on a sample of women enrolled at the hospital after delivery. Enrolled mothers were not necessarily those who had received the IBCLC intervention. Breastfeeding rates for the general population of mother-infant dyads discharged from our hospital were also calculated. In order to evaluate the IBCLC intervention, Phase III data were compared with data collected at the baseline phase (Phase I).

### 2.2. Statistical Analysis

We opted for a convenience sample and tried to enroll 200 women in Phase I and 200 in Phase III. We realized that it would have been difficult, with these numbers, to detect a reasonable significant change in the rate of exclusive breastfeeding considering that with β = 0.2 and α = 0.05, two groups of 200 women would be appropriate to detect a 12% increase in the exclusive breastfeeding rate, from 65% (our baseline) to 77%.

Data were reported as absolute frequencies and percentages. Odds ratios (pre-intervention was taken as reference) and 95% confidence intervals were used to analyze differences between before and during intervention. Differences in proportions were analyzed using a two-tailed Fisher exact test. Multivariate logistic regression models were used to adjust odds ratios for all the variables relative to the prenatal and early postnatal periods (previous BF experience, yes *vs.* no; type of delivery, cesarean sections *vs.* vaginal; attendance to birth preparation course, yes *vs.* no; skin-to-skin for at least 30 min immediately after birth, yes *vs.* no; autonomous antenatal search for information on BF, yes *vs.* no), as these factors were considered not modifiable by the intervention of IBCLC. We used a saturated model including all the variables that were unbalanced between the before and during intervention groups. A *p* < 0.05 was considered statistically significant. Analyses were carried out using Stata/IC, version 11.2 for Windows (StataCorp LP. College Station, TX, USA, 2009).

## 3. Results

We enrolled 402 mothers after childbirth, 200 during Phase I (baseline), and 202 during Phase III (during intervention). Among the 402 enrolled mothers, 391 answered the interview (response rate 97.3%) at an average of 15 days after birth (range 10–28 days).

Some differences were found between the two phases in variables concerning the prenatal and the early postnatal period. Attendance to antenatal classes and antenatal search for information on breastfeeding were significantly higher in the baseline group (Table 1). Skin-to-skin contact immediately after birth was higher in the Phase III group. The cesarean section rate was similar in the two groups (19% and 18%) (Table 1). The median length of the postpartum hospital stay was three days in both groups (defined as numbers of midnight from delivery to discharge) (interquartile range 2–4).

**Table 1:** Characteristics of the mothers.

Characteristics of the Mother	Before	During	*p*-Value *
	n (%)	n (%)	
Previous experience with breastfeeding	86 (43.0)	78 (40.8)	ns
Attendance to antenatal classes	151 (75.5)	108 (56.5)	<0.001
Antenatal autonomous search for information on breastfeeding	132 (66.0)	102 (53.4)	0.013
Cesarean delivery	39 (19.5)	34 (17.8)	ns
Skin-to-skin for at least 30 min immediately after birth	114 (57.3)	157 (82.2)	<0.001
Total	200	191	

Before: women in the pre-intervention group. During: women in the during-intervention group; ns: not significant; * *p*-value of a two-tailed Fisher exact test.

Differences between Phase I and Phase III in variables associated with breastfeeding experience are reported in Table 2. An improvement was observed in several aspects in the during-intervention group as compared to the baseline. A higher percentage of women received the correct information (explanation on breastfeeding on cue, reassurance on the benefits of breast milk, not contradicting information on breastfeeding) and support (demonstration on how to latch on and off, on how to express breast milk, on support for achieving exclusive breastfeeding). Seventy-eight percent of women in the during-intervention group started BF within two hours from delivery with a significant increase as compared to the baseline (65%, *p* = 0.004). The great majority of mothers reported a good experience with the hospital staff: the staff understood the needs of the mother, and supported the BF experience and the mother-infant relationship. The provided information regarding community groups supporting breastfeeding also significantly improved (Table 2). All of the differences were still significant after adjusting for type of delivery, attendance to antenatal courses, previous experience of BF, and skin-to-skin contact within 30 min after birth.

**Figure 1:**
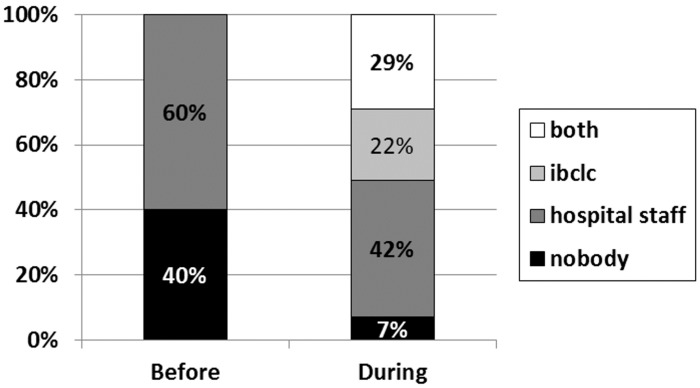
Percentage of women who received help with breastfeeding from hospital staff and or IBCLC.

The overall proportion of women who stated they received significant help with breastfeeding increased from 60% to 93% (*p* < 0.001) (Figure 1). In particular, the proportion of women who received help from the hospital staff increased, but not significantly (alone or together with IBCLC 60% *vs.* 71%, *p* = 0.09), while an additional 22% of women in the Phase III group received help only from the IBCLC.

The frequency of sore and/or cracked nipples was significantly lower in Phase III, while no difference was found in exclusive BF rates at hospital discharge or at two weeks after birth (Table 2).

The exclusive BF rate two weeks after birth was higher than that at hospital discharge, both in Phase I and Phase III.

**Table 2:** Frequencies, crude and adjusted odds ratios for during *vs.* pre-intervention responses of mothers to statements regarding education, support, and breastfeeding-related hospital practices.

Responses of Mothers	Before		During		*p*-Value			During *vs.* Before				
	n	(%)	n	(%)				Crude OR	(95% CI)	Adj OR *	(95% CI) *	*p*-Value
Hospital staff demonstrated to me how to latch on	113	(56.5)	168	(88.0)	<0.001			5.6	(3.6–9.4)	6.6	(3.7–12.2)	<0.001
Hospital staff assured me that breast milk fully responds to the needs of the baby in the first days of life	124	(62.0)	172	(90.1)	<0.001			5.5	(3.2–9.6)	5.5	(3.0–10.0)	<0.001
Hospital staff helped me give my baby only my breast milk, without any other supplementary liquid if not really needed	106	(53.0)	165	(86.4)	<0.001			5.6	(3.4–9.2)	4.8	(2.9–8.3)	<0.001
Hospital staff explained to me to breastfeed on cue, without timetable and need of fix quantities	113	(56.5)	178	(93.2)	<0.001			10.5	(5.6–19.8)	9.1	(4.7–17.5)	<0.001
Breastfeeding started in the first two hours after birth	131	(65.5)	150	(78.5)	0.004			1.9	(1.2–3.0)	1.2	(0.6–2.1)	ns
Night and day rooming-in	135	(67.5)	186	(97.4)	<0.001			16.8	(6.6–43.0)	18.8	(7.0–50.6)	<0.001
Pacifier used during hospital stay	33	(16.5)	13	(6.8)	0.003			0.4	(0.2–0.7)	0.4	(0.2–0.9)	0.022
Bottle used during hospital stay	93	(46.5)	78	(40.8)	ns			0.8	(0.5–1.2)	0.8	(0.5–1.3)	ns
Hospital staff showed me how to express breast milk	56	(28.0)	149	(78.0)	<0.001			9.1	(5.8–14.5)	10.0	(5.9–16.9)	<0.001
Hospital staff did not give me contradictory advice on breastfeeding	94	(47.0)	154	(80.6)	<0.001			4.7	(3.0–7.4)	4.2	(2.6–6.7)	<0.001
I received significant help on breastfeeding from hospital staff **	120	(60.0)	178	(93.2)	<0.001			9.1	(4.9–17.1)	9.5	(4.8–18.8)	<0.001
Hospital staff understood my needs as a new mother (“yes” or “to some extent”)	157	(78.5)	183	(95.8)	<0.001			6.3	(2.9–13.7)	5.6	(2.4–12.6)	<0.001
I felt myself forced to practice rooming-in for longer than I wished	61	(30.5)	12	(6.3)	<0.001			0.2	(0.1–0.3)	0.2	(0.1–0.3)	<0.001
Hospital staff supported my experience with breastfeeding (“yes” or “to some extent”)	143	(71.5)	184	(96.3)	<0.001			10.1	(4.5–22.9)	10.5	(4.5–24.7)	<0.001
Hospital staff supported the building of a relationship between my baby and I (“yes” or “to some extent”)	160	(80.0)	187	(97.9)	<0.001			11.7	(4.1–33.4)	12.1	(4.0–6.2)	<0.001
At Discharge from the Hospital												
I received information on the facilities in the community to contact in case of problems with breastfeeding	183	(91.5)	182	(95.3)	ns	1.9			(0.8–4.3)	1.9	(0.8–4.7)	ns
I received information on the groups in the community supporting breastfeeding mothers	22	(11.0)	149	(78.0)	<0.001	28.7			(16.4–50.2)	24.3	(13.7–43.3)	<0.001
I had sore/cracked nipples	83	(41.5)	47	(24.6)	<0.001	0.5			(0.3–0.7)	0.4	(0.2–0.6)	<0.001
Exclusive BF at discharge	130	(65.0)	120	(62.8)	ns	0.9			(0.6–1.4)	0.8	(0.5–1.4)	ns
Exclusive BF two weeks after birth	160	(80.0)	150	(78.5)	ns	0.9			(0.6–1.5)	0.8	(0.5–1.5)	ns

Before: women in the pre-intervention group; During: women interviewed during the intervention; 95% CI: 95% confidence interval; OR: odds ratio; ns: not significant; * Adjusted for: previous BF experience, yes *vs.* no; type of delivery, cesarean sections *vs.* vaginal; attendance to birth preparation course, yes *vs.* no; skin-to-skin for at least 30 min immediately after birth, yes *vs.* no; autonomous antenatal search for information on BF, yes *vs.* no; ** During baseline, hospital staff was comprised of only nurses and midwives, while during the intervention it also includes the IBCLC.

## 4. Discussion

According to our study, the inclusion of a lactation consultant (IBCLC) in the maternity ward of a mother and child hospital increased the quality of breastfeeding support, increased women’s satisfaction, and reduced the prevalence of sore/cracked nipples.

In fact, during the intervention, more mothers received basic information on breastfeeding (e.g., feeding on cue, less contradictory advice) and breastfeeding support (e.g., regarding latch-on and breast milk expression) from hospital staff. Moreover, more mothers practiced actions (e.g., night and day rooming-in, no pacifier and/or bottle) well known to contribute to the successful initiation of breastfeeding during their hospital stay.

With the support of the IBCLC, more babies benefited from skin-to-skin contact immediately after birth and were put at the breast in the first two hours after birth, although the lactation consultant did not work specifically to improve skin-to-skin, and the time she spent in the delivery room was occasional. We speculate that this change in practice might be due to factors not considered by the present study or to a cultural osmosis related to the work of an IBCLC in the postpartum ward of the same maternity.

Eventually, at hospital discharge, mothers were better-informed on the breastfeeding resources at the community level.

A second relevant result includes the significant reduction of sore/cracked nipples, from 41.5% to 24.6%. We can consequently state that the IBCLC intervention contributed to the reduction of a potential cause of BF interruption, possibly by improving the quality of the latch-on of newborns to the breast. The difference in the prevalence of sore/cracked nipples remained significant after correction for type of delivery, previous experience with BF, latch-on within two hours, and support received from hospital staff (data not shown).

Nevertheless, despite recorded improvements in breastfeeding education and practice, breastfeeding rates in the first two weeks after delivery did not increase.

The rate of exclusive breastfeeding at two weeks after birth increased if compared to the rate at hospital discharge, possibly due to different recall periods (from birth to discharge during hospital stay *vs.* the previous 24 h afterwards) in defining infant feeding categories.

Several reasons might be mentioned that could explain the lack of the significant increase in exclusive BF rates. First, we must notice that the two compared samples of mothers before and during the IBCLC intervention differ for attendance to antenatal classes and for information on breastfeeding; the lower rates during the intervention phase are probably due to a temporary gap in the availability of antenatal classes as a consequence of a sudden change in the organization of the prenatal classes, which were formerly hospital-based and later run by the community health services. Actually, this represented an unforeseeable event. Second, we should consider that the additional help for breastfeeding mothers in the maternity ward was just constituted by only one IBCLC, possibly not enough to have an impact on breastfeeding rates at discharge, as a consequence of direct and indirect (through interaction with nurses) support to mothers. In fact, according to the recommendations made by Mannel and Mannel [31] for optimal IBCLC staffing, one full-time equivalent per 783 breastfeeding couplets is required, which would represent at least two full-time equivalents for our maternity ward. Third, the effectiveness of a proactive intervention in promoting breastfeeding is well known in the literature [32]. Nevertheless, the support to breastfeeding in our study was proactive only during the hospital stay, while from hospital discharge to the telephone interview, the IBCLC was available only for on-demand telephone support in addition to the help routinely offered by community health workers. Fourth, the breastfeeding rates at discharge (nearly 70%) and at two weeks after birth (nearly 80%) were already relatively high, which could dampen the impact of an IBCLC intervention. These above-mentioned elements can be tackled by implementing health policy initiatives such as the WHO/UNICEF Ten Steps to Successful Breastfeeding [33]. Future research on the topic should involve greater study samples in order to have greater influence. Fifth, we must recognize that adding an IBCLC without changing anything else (*i.e.*, fully implementing the Ten Steps), which is certainly a limitation of our study, did not change care/behavior on the part of the rest of the staff and consequently did not modify breastfeeding rates. In fact, it is possible that more mothers received breastfeeding help simply because there was one person on staff whose sole responsibility was to provide breastfeeding support. Finally, our LC did not have a healthcare qualification, an issue that possibly caused some initial difficulties in the integration of the IBCLC with maternity nurses in our setting. In Italy, there is currently no regulated position for LCs among maternity staff, unless the LC has also a professional background. We believe that not being health professionals does not diminish the potential effectiveness of an IBCLC in the support of breastfeeding, but it certainly raises our attention in terms of potential difficult inclusion and integration into the maternity ward.

We also realize that an additional effort should be made to define the tasks of an IBCLC working with the nurse staff in order to guarantee optimal integration and more effective support for breastfeeding, and to avoid overlaps. The IBCLC in this study was basically replacing/complementing the work of the nursing staff, which may not have been the best utilization.

We should also mention that a limit to this study has been not collecting information on the age and formal education status of the mothers in order to verify if there were significant differences between the two groups. Nevertheless, statistics for the Trieste area show that the maternal average age at childbirth only slightly increased from 31.4 years in 2006 up to 31.7 years in 2008 [34]. Moreover, the two groups did not differ in terms of previous breastfeeding experience and there is no reason to believe the two groups were unbalanced because of these variables. Even if women in the baseline group were different in terms of attendance to antenatal courses and autonomous searches for information, the significant impact of the intervention remained substantially unchanged after adjusting for these factors.

In terms of sample size, as previously mentioned, for logistical reasons our sample was underpowered to detect an increase in exclusive BF of less than 12%, which is quite high if we consider that the inclusion of an IBCLC was the only intervention put in place.

We believe that a study comparing two groups, one taken before the intervention and the other taken toward the end of the intervention, when the intervention is consolidated, is a good compromise. We could have adopted other designs, but all have positive and negative aspects to be considered. Randomization was excluded for two reasons: ethical reasons, and because it would not be possible to avoid a change in attitude of the rest of the nursing staff in a maternity ward in which an IBCLC was introduced. We also excluded the allocation of two maternity wards, one to the intervention and the other with no intervention, because there are no other maternity wards in Trieste, and a comparison between two maternity wards from different hospitals and in different towns would have implied more heterogeneity problems than a pre- and post- study design.

## 5. Conclusions

We must conclude that, at least in our setting, the inclusion of an IBCLC among the staff of the maternity ward was appreciated by mothers and permitted mothers to improve their experience with breastfeeding. Moreover, it was associated with a lower prevalence of sore/cracked nipples. It did not, however, succeed in increasing the exclusive breastfeeding rate.

We believe that this study does not prove the lack of effectiveness of the intervention of an IBCLC, and instead tells us that acquiring an IBCLC without changing policies and staff practices cannot *per se* improve breastfeeding outcome, although it can have an overall positive impact.

In our opinion, an increase of the breastfeeding rate might be possible with an extended proactive intervention by IBCLCs along with adequately staffed nurses, possibly associated with the implementation of the WHO/UNICEF Ten Steps for the protection, promotion, and support of breastfeeding.
